# *Burkholderia thailandensis* Isolated from Infected Wound, Arkansas, USA

**DOI:** 10.3201/eid2411.180821

**Published:** 2018-11

**Authors:** Jay E. Gee, Mindy G. Elrod, Christopher A. Gulvik, Dirk T. Haselow, Catherine Waters, Lindy Liu, Alex R. Hoffmaster

**Affiliations:** Centers for Disease Control and Prevention, Atlanta, Georgia, USA (J.E. Gee, M.G. Elrod, C.A. Gulvik, L. Liu, A.R. Hoffmaster);; Arkansas Department of Health, Little Rock, Arkansas, USA (D.T. Haselow, C. Waters)

**Keywords:** melioidosis, Burkholderia, thailandensis, pseudomallei, clinical, bacteria, wound, Arkansas, United States

## Abstract

The bacterium *Burkholderia thailandensis*, a member of the *Burkholderia pseudomallei* complex, is generally considered nonpathogenic; however, on rare occasions, *B. thailandensis* infections have been reported. We describe a clinical isolate of *B. thailandensis*, BtAR2017, recovered from a patient with an infected wound in Arkansas, USA, in 2017.

*Burkholderia thailandensis* is a member of the *Burkholderia pseudomallei* complex and is generally considered nonpathogenic (*1*). *B. pseudomallei* causes the disease melioidosis and can be fatal even when properly treated (*1*–*3*). *B. thailandensis* was first discovered in Thailand and was differentiated from *B. pseudomallei* phenotypically by its ability to assimilate arabinose (*3*). *B. thailandensis*, like *B. pseudomallei*, naturally occurs in the environment (e.g., in moist soils) and is associated with tropical or subtropical climates (*3*,*4*). Environmental isolates of *B. thailandensis* have been predominantly recovered in Southeast Asia, with sporadic reports in other regions such as in Australia, which is considered the ancestral origin for *B. pseudomallei* based on analysis of genomic single-nucleotide polymorphisms (SNPs) (*4*–*6*).

The phenotypic similarities are substantial among members of the *B. pseudomallei* complex, other *Burkholderia* species, and other bacteria such as *Pseudomonas* spp. Laboratory personnel unfamiliar with *B. pseudomallei* often have difficulty identifying it using microbiologic methods commonly available in clinical settings. Automated biochemical systems might also misidentify *B. pseudomallei* because of an insufficient number of reference strains in their databases. Incorrect identification can delay appropriate antimicrobial therapy (*1*,*4*,*7*).

Although rare, *B. thailandensis* infections in humans have been reported. The first case reported in the literature occurred in or before 1999 in Thailand in a 16-year-old boy who suffered compound fractures in a motorcycle accident (*8*). The second case occurred in 1997 in a 76-year-old man in Louisiana, USA, who had a pleural wound; this case resulted in recovery of isolate H0587 (also known as CDC2721121). Further details on the wound and whether it was acquired locally or whether the patient had a travel history to areas endemic for *B. thailandensis* are not available. The third case occurred in 2003 in a previously healthy 2-year-old boy in Texas, USA, who aspirated water from a ditch after a car accident; this case resulted in recovery of isolate TXDOH (also known as CDC3015869) (*9*). The fourth case occurred in 2011 in a 42-year-old man in Malaysia who had a foot abscess with ankle swelling and skin cellulitis (*10*). The fifth report was a fatal case that occurred in 2013 in a 67-year-old man who was treated at a hospital in Chongquing, China (*11*); however, subsequent correspondence suggested that the species had been identified incorrectly and was most likely *B. pseudomallei* (*12*).

In April 2017, a 29-year-old woman with diabetes in Arkansas, USA, crashed into a large metal trash bin while driving an all-terrain vehicle, resulting in an open bone forearm fracture. Treatment of the wound included installation of a metal plate. Approximately 3 months later, the patient returned to the hospital because of a 2-week history of swelling of her forearm. An isolate was recovered from pure culture graded 1+ (trace growth) from a deep operative tissue specimen from the forearm wound. Initial antimicrobial treatment consisted of piperacillin/tazobactam, vancomycin, and cefazolin. Testing of the isolate using the Microscan Walkaway automated biochemical system (Beckman Coulter, Atlanta, GA, USA) by the hospital laboratory identified *B. pseudomallei*. Based on this result, the Arkansas Department of Health (DoH) contacted the Centers for Disease Control and Prevention (CDC) about a suspected case of melioidosis. After the hospital transferred the isolate to the Arkansas DoH, *B. pseudomallei* was ruled out by using biochemical and PCR testing. The isolate was presumptively identified as *B. thailandensis* by using the Bruker MALDI Biotyper mass spectrometer (Bruker Daltonics, Billerica, MA, USA). Arkansas DoH forwarded the isolate, designated BtAR2017, to CDC for confirmation and further characterization. The patient was discharged with 6 weeks of intravenous ceftazidime treatment. She last saw her healthcare provider in December 2017 and was reported completely healed.

## The Study

Biochemical testing, including arabinose assimilation, identified isolate BtAR2017 as *B. thailandensis*. We extracted DNA from the isolate for next-generation sequencing (Technical Appendix). We analyzed the genome of BtAR2017 for multilocus sequence typing (MLST), which yielded sequence type (ST) 101. ST101 was previously identified in *B. thailandensis* isolates TXDOH and H0587 (*9*). Although MLST is commonly used to subtype members of the *B. pseudomallei* complex, it has only a moderate level of resolution and, because of recombination, might not reflect actual levels of relatedness (*5*,*13*). 

For high-resolution analysis, we compared the genome of BtAR2017 with a reference panel of publicly available *B. thailandensis* genomes (Table). A dendrogram based on the SNP analysis indicates that BtAR2017 clusters with H0587 and TXDOH (Figure). Also within the BtAR2017 cluster is E555, an environmental isolate recovered in Cambodia in 2005, and strain 2.1, which metadata in the National Center for Biotechnology Information entry indicate was recovered in June 2017 from soil in Vietnam (*14*). This cluster appears as an outlier subgroup compared with other examples of *B. thailandensis,* including E264^T^ (type strain). MLST indicates that E555 is ST696, which is a single-locus variant of ST101. Comparison of the genome sequences indicates >4,700 core SNPs between BtAR2017 and E555, compared with >32,700 SNPs between BtAR2017 and E264^T^. BtAR2017 has >2,200 SNPs compared with H0587 but >5,900 SNPs compared with TXDOH.

**Table:** Reference *Burkholderia* spp. genomes used for characterization of *Burkholderia thailandensis* isolated from an infected wound, Arkansas, USA, 2017*

Isolate	Other identifiers	Origin	Source	ST†	GenBank accession nos.	ANI	SNP
82172	34; 2002721621	France	Horse (foal)	73	NZ_LNNG00000000		X
Bt4	49639	Australia	Environmental	699	NZ_ABBH00000000		X
E1		Papua New Guinea	Environmental	669	NZ_LOXF00000000		X
E254		Thailand	Environmental	345	NZ_CP004381.1; NZ_CP004382.1		X
E264	ATCC 700388	Thailand	Environmental	80	CP008785.1; CP008786.1	X	X
E444		Thailand	Environmental	79	NZ_CP004117.1; NZ_CP004118.1		X
E555		Cambodia	Environmental	696	NZ_AECN00000000		X
H0587	BtCDC2721121; 2002721121	USA (Louisiana)	Human	101	NZ_CP013409.1; NZ_CP013410.1		X
K96243		Thailand	Human	10	NC_006350.1; NC_006351.1	X	
MSMB59	MSMB0059	Australia	Environmental	699	NZ_CP004385.1; NZ_CP004386.1		X
MSMB60	MSMB0060	Australia	Environmental	699	NZ_LOXG00000000		X
Phuket 4W-1		Thailand	Environmental	80	NZ_AQQJ01000000		X
Strain 2.1		Vietnam	Environmental	696	PHRD00000000		X
TXDOH	CDC3015869; 2003015869	USA (Texas)	Human	101	NZ_CP013360.1; NZ_CP013361.1	X	X
USAMRU Malaysia no. 20	Malaysia #20; 2002721744	Malaysia	Unknown	80	NZ_CP004383.1; NZ_CP004384.1		X

*X indicates inclusion in analysis. ANI, average nucleotide identity; SNP, single nucleotide polymorphism; ST, sequence type.  †By multilocus sequence typing.

**Figure:**
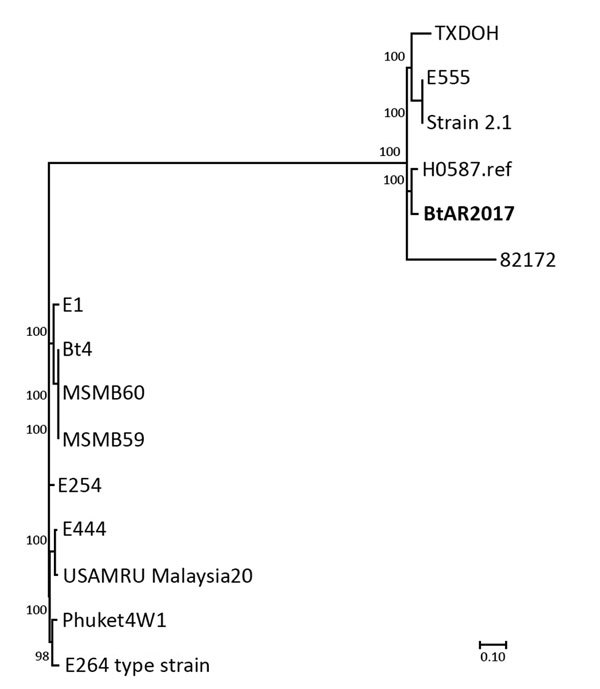
Dendrogram used for characterization of *Burkholderia thailandensis* isolate (bold) from an infected wound, Arkansas, USA, 2017; compare, with reference isolates. Generated in MEGA 7.0 software (http://www.megasoftware.net) from results of maximum-parsimony phylogenetic analysis of core single-nucleotide polymorphisms from available *B. thailandensis* genomes (conducted by using Parsnp, a component of the Harvest 1.3 software suite [https://github.com/marbl/harvest]). Scale bar indicates number of substitutions per SNP.

Sim et al. (*14*) previously noted this outlier subgroup and postulated that its members, including E555, might be more virulent than typical examples of *B. thailandensis*. However, their studies challenging BALB/c mice and the nematode *Caenorhabditis elegans* with E555 did not reveal any significant difference in virulence compared with E264^T^ (*14*). Another study by Deshazer using TXDOH and H0587 to challenge mice and hamsters indicated that in these models TXDOH also had a similar level of virulence as E264^T^, whereas H0587 was avirulent (*15*). E555 is known to have features such as a *B. pseudomallei*–like capsular polysaccharide (*14*). We tested BtAR2017 for *B. pseudomallei* capsular polysaccharide, but results were negative.

We analyzed average nucleotide identity (ANI) and determined that the genome for BtAR2017 has 98.99% identity with that of E264^T^, which is above the 95% threshold commonly used for distinguishing species. The BtAR2017 genome also had an ANI of 99.75% compared with TXDOH but only 92.7% compared with K96243, a representative *B. pseudomallei* strain used in lieu of the species type strain. Although BtAR2017 is in the outlier subgroup resolved by genomic SNP analysis, these ANI results suggest that BtAR2017 and other members of the subgroup are *B. thailandensis* and not a novel species.

## Conclusions

The recovery of the clinical isolates suggests that *B. thailandensis* might be endemic to the continental United States and capable of causing opportunistic infections associated with traumatic injuries. Because of the limited number of reports, this bacterium’s geographic range in North America is unknown. Two of the 3 clinical isolates recovered in the United States are now well documented and were acquired in the southern states of Texas and Arkansas, possibly reflecting the affinity for warm climates associated with most members of the *B. pseudomallei* complex (the exception being *B. mallei*, which is host-adapted to equids and does not persist in the environment). These 3 clinical isolates also are members of the outlier subgroup on the dendrogram (Figure), which suggests a genetic bottleneck or discrete seeding event for the United States, assuming the Louisiana case was acquired locally. Further study of *B. thailandensis* will improve our knowledge of its geographic range and ability to cause infections.

Technical Appendix Materials and methods used for characterization of *Burkholderia thailandensis* isolated from an infected wound, Arkansas, USA, 2017.

## References

[1] Tuanyok A, Mayo M, Scholz H, Hall CM, Allender CJ, Kaestli M, et al. *Burkholderia humptydooensis* sp. nov., a new species related to *Burkholderia thailandensis* and the fifth member of the *Burkholderia pseudomallei* complex. Appl Environ Microbiol. 2017;83:e02802–16. 10.1128/AEM.02802-16 27986727 PMC5311406

[2] Wiersinga WJ, Virk HS, Torres AG, Currie BJ, Peacock SJ, Dance DAB, et al. Melioidosis. Nat Rev Dis Primers. 2018;4:17107. 10.1038/nrdp.2017.107 29388572 PMC6456913

[3] Brett PJ, DeShazer D, Woods DE. *Burkholderia thailandensis* sp. nov., a *Burkholderia pseudomallei*-like species. Int J Syst Bacteriol. 1998;48:317–20. 10.1099/00207713-48-1-317 9542103

[4] Levy A, Merritt AJ, Aravena-Roman M, Hodge MM, Inglis TJ. Expanded range of *Burkholderia* species in Australia. Am J Trop Med Hyg. 2008;78:599–604. 10.4269/ajtmh.2008.78.599 18385355

[5] Pearson T, Giffard P, Beckstrom-Sternberg S, Auerbach R, Hornstra H, Tuanyok A, et al. Phylogeographic reconstruction of a bacterial species with high levels of lateral gene transfer. BMC Biol. 2009;7:78. 10.1186/1741-7007-7-78 19922616 PMC2784454

[6] Ginther JL, Mayo M, Warrington SD, Kaestli M, Mullins T, Wagner DM, et al. Identification of *Burkholderia pseudomallei* near-neighbor species in the Northern Territory of Australia. PLoS Negl Trop Dis. 2015;9:e0003892. 10.1371/journal.pntd.0003892 26121041 PMC4486726

[7] Hoffmaster AR, AuCoin D, Baccam P, Baggett HC, Baird R, Bhengsri S, et al. Melioidosis diagnostic workshop, 2013. Emerg Infect Dis. 2015;21:21. 25626057 10.3201/eid2102.141045 PMC4313648

[8] Lertpatanasuwan N, Sermsri K, Petkaseam A, Trakulsomboon S, Thamlikitkul V, Suputtamongkol Y. Arabinose-positive *Burkholderia pseudomallei* infection in humans: case report. Clin Infect Dis. 1999;28:927–8. 10.1086/517253 10825075

[9] Glass MB, Gee JE, Steigerwalt AG, Cavuoti D, Barton T, Hardy RD, et al. Pneumonia and septicemia caused by *Burkholderia thailandensis* in the United States. J Clin Microbiol. 2006;44:4601–4. 10.1128/JCM.01585-06 17050819 PMC1698378

[10] Zueter AM, Abumarzouq M, Yusof MI, Wan Ismail WF, Harun A. Osteoarticular and soft-tissue melioidosis in Malaysia: clinical characteristics and molecular typing of the causative agent. J Infect Dev Ctries. 2017;11:28–33. 10.3855/jidc.7612 28141587

[11] Chang K, Luo J, Xu H, Li M, Zhang F, Li J, et al. Human infection with *Burkholderia thailandensis*, China, 2013. Emerg Infect Dis. 2017;23:1416–8. 10.3201/eid2308.170048 28726626 PMC5547772

[12] Dance DAB, Sarovich D, Price EP, Limmathurotsakul D, Currie BJ. Human Infection with *Burkholderia thailandensis*, China, 2013. Emerg Infect Dis. 2018;24:953–4. 10.3201/eid2405.180238 29664392 PMC5938794

[13] Gee JE, Gulvik CA, Elrod MG, Batra D, Rowe LA, Sheth M, et al. Phylogeography of *Burkholderia pseudomallei* Isolates, Western Hemisphere. Emerg Infect Dis. 2017;23:1133–8. 10.3201/eid2307.161978 28628442 PMC5512505

[14] Sim BM, Chantratita N, Ooi WF, Nandi T, Tewhey R, Wuthiekanun V, et al. Genomic acquisition of a capsular polysaccharide virulence cluster by non-pathogenic *Burkholderia* isolates. Genome Biol. 2010;11:R89. 10.1186/gb-2010-11-8-r89 20799932 PMC2945791

[15] Deshazer D. Virulence of clinical and environmental isolates of *Burkholderia oklahomensis* and *Burkholderia thailandensis* in hamsters and mice. FEMS Microbiol Lett. 2007;277:64–9. 10.1111/j.1574-6968.2007.00946.x 17986086

