# Supplementary material for: Burkholderia thailandensis Isolated from Infected Wound, Arkansas, USA
Source: Emerg Infect Dis. 2018 Nov;24(11):2091–4. doi: 10.3201/eid2411.180821 (PMC6199988; doi:10.3201/eid2411.180821)
Supplement: Technical Appendix — Materials and methods used for characterization of Burkholderia thailandensis isolated from an infected wound, Arkansas, USA, 2017. [file 18-0821-Techapp-s1.pdf]

Article DOI: https://doi.org/10.3201/eid2411.180821

Burkholderia thailandensis Isolated from Infected Wound, Arkansas, USA

Technical Appendix

Materials and Methods

DNA was extracted using the Maxwell RSC Cultured Cells DNA kit on the Promega Maxwell RSC Instrument per the manufacturer's instructions (Promega, Fitchburg, Wisconsin). The sample was eluted in PCR-grade water and RNase A, filtered through a 0.1 µm filter, and checked for sterility before submission for whole genome sequencing (*1*).

The sequence for BtAR2017 was determined from paired-end Illumina reads which were generated on an Illumina MiSeq (Illumina, Inc., San Diego, California). Genomic DNA was sheared to a mean size of 600 bp using a Covaris LE220 focused ultrasonicator (Covaris Inc., Woburn, MA). DNA fragments were Ampure (Beckman Coulter Inc., Indianapolis, IN) cleaned and used to prepare dual-indexed sequencing libraries using NEBNext Ultra library prep reagents (New England Biolabs Inc., Ipswich, MA) and barcoding indices synthesized in the CDC Biotechnology Core Facility. Libraries were analyzed for size and concentration, pooled and denatured for loading onto flowcell for cluster generation. Sequencing was performed on the Illumina Miseq using 2 × 250 bp cycle paired-end sequencing kits. On completion, sequence reads were filtered for read quality, basecalled and demultiplexed using bcl2fastq (v2.19). Raw read data were archived at NCBI as SRA accession SRP135864.

The genome was submitted to the *Burkholderia pseudomallei* MLST Web site (http://pubmlst.org/bpseudomallei) to identify the sequence type (*2*,*3*). Core SNPs were analyzed using Parsnp in the Harvest 1.3 suite (*4*). The dendrogram was generated in MEGA7 (*5*). Average nucleotide Identity (ANI) was calculated at http://enve-omics.ce.gatech.edu/ani/index (*6*). The Active Melioidosis Detect kit (inBios, Seattle, WA) was used to test BtAR2017 for the presence of *B. pseudomallei* capsular polysaccharide as per manufacturer's instructions (*7*).

References

1. Gee JE, Gulvik CA, Elrod MG, Batra D, Rowe LA, Sheth M, et al. Phylogeography of *Burkholderia pseudomallei* isolates, Western Hemisphere. Emerg Infect Dis. 2017;23:1133–8. PubMed http://dx.doi.org/10.3201/eid2307.161978

2. Jolley KA, Maiden MC. BIGSdb: scalable analysis of bacterial genome variation at the population level. BMC Bioinformatics. 2010;11:595. PubMed http://dx.doi.org/10.1186/1471-2105-11-595

3. Godoy D, Randle G, Simpson AJ, Aanensen DM, Pitt TL, Kinoshita R, et al. Multilocus sequence typing and evolutionary relationships among the causative agents of melioidosis and glanders, *Burkholderia pseudomallei* and *Burkholderia mallei*. J Clin Microbiol. 2003;41:2068–79. PubMed http://dx.doi.org/10.1128/JCM.41.5.2068-2079.2003

4. Treangen TJ, Ondov BD, Koren S, Phillippy AM. The Harvest suite for rapid core-genome alignment and visualization of thousands of intraspecific microbial genomes. Genome Biol. 2014;15:524. PubMed http://dx.doi.org/10.1186/s13059-014-0524-x

5. Kumar S, Stecher G, Tamura K. MEGA7: Molecular Evolutionary Genetics Analysis Version 7.0 for bigger datasets. Mol Biol Evol. 2016;33:1870–4. PubMed http://dx.doi.org/10.1093/molbev/msw054

6. Goris J, Konstantinidis KT, Klappenbach JA, Coenye T, Vandamme P, Tiedje JM. DNA-DNA hybridization values and their relationship to whole-genome sequence similarities. Int J Syst Evol Microbiol. 2007;57:81–91. PubMed http://dx.doi.org/10.1099/ijs.0.64483-0

7. Houghton RL, Reed DE, Hubbard MA, Dillon MJ, Chen H, Currie BJ, et al. Development of a prototype lateral flow immunoassay (LFI) for the rapid diagnosis of melioidosis. PLoS Negl Trop Dis. 2014;8:e2727. PubMed http://dx.doi.org/10.1371/journal.pntd.0002727
